# Supplementary material for: A Critical View of the Application of the APEX Software (Aqueous Photochemistry of Environmentally-Occurring Xenobiotics) to Predict Photoreaction Kinetics in Surface Freshwaters
Source: Molecules. 2019 Dec 18;25(1):9. doi: 10.3390/molecules25010009 (PMC7017383; doi:10.3390/molecules25010009)
Supplement: Supplementary file 1 [file molecules-25-00009-s001.zip › 3.2.4_gcc-4.4.0.zip!/3.2.4_gcc-4.4.0/3.2.4_gcc-4.4.0/doc/octave/html/interpreter/C_002b_002b-Sources.html]

C++ Sources - Untitled


Next: Other Sources,
Previous: Octave Sources (m-files),
Up: Contributing Guidelines


---

### D.4 C++ Sources

Don't use tabs. Tabs cause trouble. If you are used to them, set up your editor
so that it converts tabs to spaces. Format function headers like this:

```
     static bool
     matches_patterns (const string_vector& patterns, int pat_idx,
     		  int num_pat, const std::string& name)
```

The function name should start in column 1, and multi-line argument lists should
be aligned on the first char after the open parenthesis. You should put a space
after the left open parenthesis and after commas, for both function definitions
and function calls.

Recommended indent is 2 spaces. When indenting, indent the statement after
control structures (like `if`, `while`, etc.). If there is a compound
statement, indent *both* the curly braces and the body of the statement (so
that the body gets indented by *two* indents). Example:

```
     if (have_args)
       {
         idx.push_back (first_args);
         have_args = false;
       }
     else
       idx.push_back (make_value_list (*p_args, *p_arg_nm, &tmp));
```

If you have nested `if` statements, use extra braces for extra
clarification.

Split long expressions in such a way that a continuation line starts with an
operator rather than identifier. If the split occurs inside braces, continuation
should be aligned with the first char after the innermost braces enclosing the
split. Example:

```
     SVD::type type = ((nargout == 0 || nargout == 1)
                       ? SVD::sigma_only
                       : (nargin == 2) ? SVD::economy : SVD::std);
```

Consider putting extra braces around a multiline expression to make it more
readable, even if they are not necessary. Also, do not hesitate to put extra
braces anywhere if it improves clarity.

Try declaring variables just before they're needed. Use local variables of
blocks - it helps optimization. Don't write multi-line variable declaration
with a single type specification and multiple variables. If the variables don't
fit on single line, repeat the type specification. Example:

```
     octave_value retval;
     
     octave_idx_type nr = b.rows ();
     octave_idx_type nc = b.cols ();
     
     double d1, d2;
```

Use lowercase names if possible. Uppercase is acceptable for variable names
consisting of 1-2 letters. Do not use mixed case names.

Try to use Octave's types and classes if possible. Otherwise, try to use C++
standard library. Use of STL containers and algorithms is encouraged. Use
templates wisely to reduce code duplication. Avoid comma expressions, labels
and gotos, and explicit typecasts. If you need to typecast, use the modern C++
casting operators. In functions, try to reduce the number of `return`
statements - use nested `if` statements if possible.
